# Breast cancer prognosis by combinatorial analysis of gene expression data

**DOI:** 10.1186/bcr1512

**Published:** 2006-07-19

**Authors:** Gabriela Alexe, Sorin Alexe, David E Axelrod, Tibérius O Bonates, Irina I Lozina, Michael Reiss, Peter L Hammer

**Affiliations:** 1 RUTCOR (Rutgers University Center for Operations Research), Piscataway, New Jersey, USA; 2 Computational Biology Center, TJ Watson IBM Research, Yorktown Heights, New York, USA; 3 The Simons Center for Systems Biology, Institute for Advanced Study, Princeton, New Jersey, USA; 4 Department of Genetics, Rutgers University, Piscataway, New Jersey, USA; 5 The Cancer Institute of New Jersey, New Brunswick, New Jersey, USA; 6 Division of Medical Oncology, UMDNJ-Robert Wood Johnson Medical School, New Brunswick, New Jersey, USA

## Abstract

**Introduction:**

The potential of applying data analysis tools to microarray data for diagnosis and prognosis is illustrated on the recent breast cancer dataset of van 't Veer and coworkers. We re-examine that dataset using the novel technique of logical analysis of data (LAD), with the double objective of discovering patterns characteristic for cases with good or poor outcome, using them for accurate and justifiable predictions; and deriving novel information about the role of genes, the existence of special classes of cases, and other factors.

**Method:**

Data were analyzed using the combinatorics and optimization-based method of LAD, recently shown to provide highly accurate diagnostic and prognostic systems in cardiology, cancer proteomics, hematology, pulmonology, and other disciplines.

**Results:**

LAD identified a subset of 17 of the 25,000 genes, capable of fully distinguishing between patients with poor, respectively good prognoses. An extensive list of 'patterns' or 'combinatorial biomarkers' (that is, combinations of genes and limitations on their expression levels) was generated, and 40 patterns were used to create a prognostic system, shown to have 100% and 92.9% weighted accuracy on the training and test sets, respectively. The prognostic system uses fewer genes than other methods, and has similar or better accuracy than those reported in other studies. Out of the 17 genes identified by LAD, three (respectively, five) were shown to play a significant role in determining poor (respectively, good) prognosis. Two new classes of patients (described by similar sets of covering patterns, gene expression ranges, and clinical features) were discovered. As a by-product of the study, it is shown that the training and the test sets of van 't Veer have differing characteristics.

**Conclusion:**

The study shows that LAD provides an accurate and fully explanatory prognostic system for breast cancer using genomic data (that is, a system that, in addition to predicting good or poor prognosis, provides an individualized explanation of the reasons for that prognosis for each patient). Moreover, the LAD model provides valuable insights into the roles of individual and combinatorial biomarkers, allows the discovery of new classes of patients, and generates a vast library of biomedical research hypotheses.

## Introduction

Microarray gene expression technology has provided extensive datasets that describe patients with cancer in a new way. Several methodologies have been used to extract information from these datasets. In this study we used the methodology of logical analysis of data (LAD) [1,2] to reanalyze the publicly available microarray dataset reported by van 't Veer and coworkers [3]. The motivation for using yet another method to analyze these data was the expectation that the specific aspects of LAD, and especially the combinatorial nature of its approach, would allow the extraction of new information on the problem of metastasis-free survival of breast cancer patients, and in particular on the role of various significant combinations of genes that may have an influence on this outcome.

The main goal of the study by van 't Veer and coworkers was to predict the clinical outcome of breast cancer (that is, to identify those patients who will develop metastases within 5 years) based on analysis of gene expression signatures. The crucial importance of this problem arises from the fact that the available adjuvant (chemo or hormone) therapy, which reduces by about one-third the risk for distant metastases, is not really necessary for 70–80% of the patients who currently receive it. Moreover, this therapy can have serious side effects and involves high medical costs. The study by van 't Veer and coworkers illustrates clearly that machine learning techniques, data mining, and other new techniques applied to DNA microarray analysis can outperform most clinical predictors currently in use for breast cancer. The study concludes that the new findings, '... provide a strategy to select patients who would benefit from adjuvant therapy'.

A specific feature of datasets coming from genomics is the presence of a very large number of measurements concerning gene expressions but only a relatively small number of observations. For instance, the attributes in the van 't Veer study correspond to more than 25,000 human genes, whereas the number of cases was only 97. In that dataset, each case is described by the expression levels of 25,000 genes, as measured by fluorescence intensities of RNA hybridized to microarrays of oligonucleotides. The cases included in the dataset are 97 lymph-node-negative breast cancer patients, who are grouped into a training set of 78 and a test set of 19 cases. The training set includes 34 positive cases (having a 'poor prognosis' signature; that is, having fewer than 5 years of metastasis-free survival) and 44 negative cases (having a 'good prognosis' signature; i.e. having more than 5 years of metastasis-free survival). The test set includes 12 positive and seven negative cases.

The van 't Veer study used DNA microarray analysis in primary breast tumors, and "applied supervised classification to identify gene expression signature strongly predictive of a short interval to distant metastases ('poor prognosis' signature) in patients without tumor cells in local lymph nodes at diagnosis (lymph node negative)". The study identified 231 genes as being significant markers of metastases, all of whose correlations with outcome exceeded 0.3 in absolute value, and it constructed an optimal prognosis classifier based on the best 70 genes. In the training set the system predicted correctly the class of 65 of the 78 cases (that is, with an accuracy of 83.3%, corresponding to a weighted accuracy of 83.6%), whereas in the test set it predicted correctly the class of 17 of the 19 cases (that is, with an accuracy of 89.5%, corresponding to a weighted accuracy of 88.7%). Weighted accuracy is defined as the average of the proportion of correctly predicted cases within the set of positive cases and that of correctly predicted negative cases in the dataset.

Numerous statistical and machine learning methods have been successfully applied to the analysis of microarray datasets; these methods include cluster analysis (hierarchical clustering [4-7], self-organizing maps [8-10], and two-way clustering [11]), regression analysis [12], nearest neighborhood methods [14], decision trees [14-17], artificial neural networks [18,19], support vector machines [20-23], principal component analysis [24-28], singular value decomposition [29-32], and multidimensional scaling [33,34]. A pattern-based recognition method has been developed using other kinds of data for prediction of outcome in preclinical and clinical trials of cancer patients [35,36].

The present study uses LAD, a combinatorics, optimization, and logic based methodology for the analysis of data. Specific features of the LAD approach include the exhaustive examination of the entire set of genes (without excluding those that have low statistical correlations with the outcome, or those that have low expression levels), focusing on the classification power of combinations of genes (without confining attention only to individual genes) and on the possibility of extracting novel information on the role of genes and of combinations of genes through the analysis of these exhaustive lists.

LAD has been shown to offer important insights into problems ranging from oil exploration [2], labor productivity analysis [37] and country creditworthiness evaluation [38], to medical application (for example, risk evaluation among cardiac patients [39,40]), polymer design for artificial bones [41], computerized pulmonology [42], genomic-based diagnosis and prognosis of lymphoma [43], and proteomics-based ovarian cancer diagnosis [44].

The present study uses LAD to analyze a breast cancer genomic dataset [3]. Our goals in re-examining that dataset are to evaluate the potential of LAD in developing a prognostic system for breast cancer using genomic data; to derive additional information about the influence of certain genes and combinations of genes; and to identify new classes of patients.

We present an introduction to LAD, and develop a new type of classification model that can distinguish between patients who will have a metastasis-free survival of 5 years from the others. The structure of the paper is as follows. In the Materials and method section we briefly present the concepts and methodology of LAD, illustrating them on a small 'demonstration model', which can distinguish between poor and good prognosis based on the expression levels of six genes. In the Results section we present an 'enhanced model' with improved accuracy, involving 17 genes and having excellent sensitivity and specificity both on the training and on the test sets. It is shown that this model distinguishes between positive and negative cases in the training set with a weighted accuracy of 100%, and exhibits a weighted accuracy of 82.5% in cross-validation experiments. On the test set, the model classifies correctly 18 out of 19 cases. Numerous other findings concerning the influence of various genes, and differences discovered between the structures of the training and the test sets are also presented in the Results section.

The presentation of the 'enhanced model' not only allows the construction of a high-accuracy prognostic model, but it also makes possible the derivation of interesting conclusions about the dataset, about significant genes and combinations of genes, and about new classes of patients, among other factors.

## Materials and methods

### LAD concepts

It can be expected that 'large' or 'small' values of the expression levels of certain genes can determine the poor or bad prognosis of a breast cancer patient. In order to express such relations in more precise terms, it is natural to replace terms such as 'large' and 'small' with conditions of the type '... is more than' or '... is less than' a certain value. It is therefore natural to examine the role of well chosen cut points associated with the expression levels of genes. For instance, the observation that low intensity levels of gene Contig15031_RC are (more or less) characteristic for a poor prognosis is imprecise; it can be reformulated as the ultra-simplistic classification system, 'If the intensity level of gene Contig15031_RC is at most 0.055 then the patient has a poor prognosis'. The assumption of this rule is valid for 25 positive and 11 negative cases in the training set (that is, it has a sensitivity of 25/34 = 73.5% and a specificity of 33/44 = 75%).

Combinations of such cut point based conditions naturally extend this idea. For instance, the combined requirement of satisfying simultaneously the three conditions 'The intensity level of gene Contig15031_RC is at most 0.055' and 'The intensity level of gene NM_004035 is at least -0.106' and 'The intensity level of the gene NM_003239 is at most -0.014' is fulfilled by 22 of the 34 positive cases in the dataset and by none of the negative ones. Again, these three requirements could be viewed as a classification system of poor prognosis cases, having a sensitivity of 64.7% and a specificity of 100%.

Such ideas are at the foundation of LAD. The essence of LAD is to detect patterns, or combinatorial biomarkers (that is, simple classifiers consisting of restrictions imposed on the values of the expression levels of the intensities of a combination of several genes); to generate patterns exhaustively and in an algorithmically efficient way; to use the collection of patterns as a prognostic system and thoroughly validate it; to extract from this collection as much additional information as possible about the role and nature of genes in the dataset (that is, to detect promoters and blockers); and to study the common characteristics of groups of patients that satisfy similar patterns.

We describe below the basic concepts used in LAD, including some of its computational aspects. In particular, we describe more precisely the concepts of support sets, patterns, pandects, and LAD-based classification systems, and we discuss the validation techniques used.

#### Cut points and binarization

One of the underlying principles of LAD is to disregard the exact values of a variable (for example, a gene), specifying for each patient only whether the corresponding value of this variable is sufficiently 'large' or 'small'. The implementation of this principle requires the determination of several cutpoints *c'*_*j*_, *c"*_*j*_, ..., for intensity levels *I*_*j *_of each gene *j*, such that the conditions requiring that the expression levels of the gene's intensity are low (or high) can be formalized as *I*_*j *_≤ *c'*_*j *_(*I*_*j *_≥ *c"*_*j*_), and so on.

LAD associates to each variable *x*_*j *_and each possible cutpoint *c*_*j *_a binary variable *y*_*j *_that is equal to 1 whenever *x*_*j *_≥ *c*_*j*_, and to 0 otherwise. In this way, a numerical variable (for example, specifying the expression levels of the intensity of a gene *j*) is transformed into a large number of binary variables. Because the size of the dataset (which has been very large from the beginning) increases even further, this problem is handled by carrying out a 'filtering' process, which retains only a 'support set' consisting of a very small number of these variables.

#### Support sets

In order to distinguish between measurements of good and of poor prognosis patients, only a tiny fraction of the information contained in the (original or binarized) dataset is needed. In particular, all of the information about the vast majority of the genes in the dataset is redundant. Moreover, even for the genes that are not redundant, only a few (usually only one) of the corresponding binary variables are needed. A set of binary variables that are sufficient to distinguish poor from good prognosis cases will be called a support set. A support set is called 'minimal' if none of its proper subsets is a support set; clearly, not every minimal support set is of minimum size. It is important to note that a dataset may admit hundreds or thousands of minimal support sets. The reduction of a large dataset to a substantially smaller one that includes only the variables in the chosen support set allows a major simplification of the problem, and has great importance for diagnosis and prognosis (although, in some cases, the presence of a limited number of redundant variables may be acceptable in terms of ensuring greater stability of results).

The problem of finding minimal support sets has been modeled elsewhere [1,2,45] as a typical 'set-covering' problem, and numerous methods are known in combinatorial optimization for the solution of this problem. In our case, the excessive dimensions of the associated set-covering problem (approximately 20,000 constraints involving between 2 and 3 million 0–1 variables) required the use of powerful heuristics to trim down the size of the problem. In order to be able to handle the large problems typical for genomic and proteomic datasets, a general heuristic size-reduction procedure has been developed [46]. The essence of this method is to balance the conflicting criteria of minimizing size and maximizing discrimination between positive and negative observations. In contrast to many statistically based methods, the support set generation procedures of LAD are guided by the collective strength of the subsets of variables, without being necessarily restricted to those variables that have the highest individual correlation coefficients with the outcome.

The feature selection procedure [46] applied for the van 't Veer dataset consists of two stages. In a first 'filtering' stage, a relatively small subset of relevant features was identified on the basis of several combinatorial, statistical, and information/theoretical criteria (for example, separation measure, envelope eccentricity, system entropy, signal to noise ratio). In the second stage, the importance of variables selected in the first step was evaluated based on the frequency of their participation in the set of all maximal patterns (see below) and generated using an efficient, total polynomial time algorithm [47], and a large proportion of the low impact variables was eliminated. This step was applied iteratively, until a Pareto-optimal support set was arrived at, which balanced the conflicting criteria of simplicity and accuracy; in the construction of the demonstration and enhanced models this support set consisted of only 6, respectively 17, of the 25,000 genes.

The high sensitivity and specificity of the prognostic system built on these small sets of genes are to a large extent due to the qualities of the underlying support set.

#### Logical patterns

A 'conjunction' is a set of conditions that require that the binary variables appearing in a selected subset of the support set take specific (0 or 1) values (that is, that the expression levels of the corresponding genes should be below or above certain cut points). The typical conjunctions appearing in most data analysis studies fix the values of not more than two or three binary variables. A conjunction is called a positive (or negative) pattern if its set of conditions are satisfied simultaneously by 'sufficiently many' of the positive (or negative) cases, and by 'sufficiently few' of the negative (or positive) cases.

For example, in the van 't Veer breast cancer dataset, if 'sufficiently many' is defined as 'at least 30%', then the three conditions 'The intensity level of the gene Contig15031_RC is at most 0.055' and 'The intensity level of the gene NM_004035 is at least -0.106' and 'The intensity level of the gene NM_003239 is at most -0.014' are fulfilled by 22 of the 34 positive cases in the training set and by none of the negative cases. Therefore, the simultaneous fulfillment of these three conditions describes a positive pattern (to be denoted P1). Similarly, the three conditions 'The intensity level of the AF018081 is at most 0.071' and 'The intensity level of the gene Contig26768_RC is at most 0.098' and 'The intensity level of the gene Contig15031_RC is at least 0.0915' are fulfilled by 15 of the 44 negative cases in the dataset and by none of the positive cases. Therefore, the simultaneous fulfillment of these three conditions describes a negative pattern (to be denoted N1).

Two of the most important characteristics of a pattern are its 'degree' and its 'coverage'. The degree of a pattern is simply the number of variables (genes) involved in its defining conditions. In our example, both P1 and N1 have degree 3. A case C is said to 'display' a pattern, or to be 'covered' by it, if the corresponding intensity levels of the gene expressions satisfy the defining conditions of that pattern. The prevalence of a positive (or negative) pattern is simply the proportion of positive (or negative) cases covered by it. For example, the three defining conditions of P1 are satisfied simultaneously by 22 of the 34 positive cases (that is, the prevalence of P1 is 64.7%). Similarly, N1 covers 15 of the 44 control cases (that is, its prevalence is 34.1%). Patterns that cover only positive or only negative cases are called 'pure' patterns. Clearly, both P1 and N1 are pure patterns. Usually, datasets that admit pure patterns of low degrees and high prevalences allow the construction of reliable LAD diagnostic and prognostic systems.

Several combinatorial algorithms [47-50] are available for the efficient generation of libraries of patterns. These pattern extraction algorithms are intended to identify exhaustively the collections of positive and negative patterns hidden in the dataset, without any prior knowledge of the distribution of the data domain.

As an indication of their efficiency, we note that the generation of the 133,920 potential patterns examined for this study and the selection of the 385 maximal pure patterns required a total computer time of 5.1 s.

It should be noted that the concept of patterns resembles that of rules, which appears in expert systems and in various decision tree-based methods. It should also be mentioned that the number of rules in a dataset is exponentially large, and therefore the generation of every possible rule is not realistic. Although most of the rule-based methods generate a relatively small number of potentially significant rules, one of the major characteristics of LAD is the systematic generation of an extremely large collection of potentially significant rules, and in a subsequent stage the 'filtering' of this collection in order to retain only a reasonably sized collection that can jointly explain the positive or negative nature of every case in the dataset. This approach not only ensures that there is the possibility of selecting those rules or patterns that, taken individually, carry the greatest amount of information (for example, have low degrees and high coverages); it also maximizes the collective inference power of the selected family of patterns. In essence, the pattern generation system of LAD consists of a systematic, exhaustive combinatorial enumeration process, which is guided by clear optimization criteria.

#### Pandects

The pandect (i.e. the collection of all of the positive and negative patterns corresponding to a dataset) is an important component of LAD because it allows construction of diagnostic and prognostic systems, analysis of the importance and role of variables, and identification of new classes of observations, among other factors. In view of the enormous number of patterns corresponding to a dataset, the construction of the entire pandect is not realistic. However, it has been seen in numerous case studies that the knowledge of special subsets of the pandect is sufficient for accurate analysis of datasets. The set of all positive (or negative) patterns of degree at most *d*^+ ^(or *d*^-^) and prevalence at least *p*^+ ^(or *p*^-^) is called the (*d*^+^, *p*^+^) positive pandect (or the (*d*^-^, *p*^-^) negative pandect). The best pandect-defining parameters *d*^+^, *d*^-^, *p*^+^, and *p*^- ^for the analysis of a particular dataset are determined experimentally by carrying out a series of *k*-fold cross-validation experiments. The computational complexity of generating the pandect depends mostly on the values of *d*^+ ^and *d*^-^. Because in most cases very small values (usually not more than 2 or 3) of *d*^+ ^and *d*^- ^are sufficient for the generation of an extremely useful pandect, this component of LAD can be calculated in a very efficient way. The particular pandect used in the present study is defined by *d*^+ ^= *d*^- ^= 3 and *p*^+ ^= *p*^- ^= 15%, and consists of 215 positive and 170 negative patterns. Although patterns can be viewed as tests that are indicative of a good or bad prognosis, the 'pandect' plays the role of a high powered prognostic battery of tests. Clearly, the pandect is not a minimal system because it may contain many redundant patterns, without which the system can still remain accurate. As a matter of fact the pandect of the van 't Veer dataset contains several minimal separating subsets of patterns (called 'models'); two such models are discussed in this report: a 'demonstration model' consisting of nine positive and seven negative patterns, and an 'enhanced model' consisting of 20 positive and 20 negative patterns. It should be added that the built-in redundancy of the large pandect of 215 + 170 patterns can substantially increase [51] the prognostic system's 'stability' or 'robustness' when it is applied to new cases.

#### Pattern space

In the given dataset, each patient is described in terms of approximately 25,000 attributes (genes) by specifying their respective expression levels. Taking into account the fact that LAD patterns can be viewed as logically synthesized attributes that can be expected to reflect more closely the condition of a patient than the original 'raw data', it is reasonable to assume that a description of patients specifying exactly the set of patterns by each individual should represent more precisely the patient's condition. This pattern-based representation of the observations can be achieved by associating to each patient and to each pattern in the pandect an indicator variable that shows whether the patient satisfies (indicator = 1) or does not satisfy (indicator = 0) the conditions that define that pattern. In this way, each patient is characterized by a sequence of 0–1 values of the indicator variables associated with the positive and negative patterns in the pandect.

#### Calibration

The quality of the prognosis given by the pandect is a consequence of the choice of several control parameters. The collection of control parameters include the number of cutpoints per gene, upper bounds on the size of support sets, pattern degrees, and lower bounds on pattern prevalence. The control parameters define uniquely the pandect. The best values of the control parameters are determined iteratively by assigning some values to them, constructing the associated pandect, verifying the correctness of its predictions, reassigning the values, and continuing this sequence of steps until one arrives at a pandect with highly accurate predictions. The verification process is based on well known statistical cross-validation techniques.

The most frequently used cross-validation techniques are the leave-one-out (or jackknifing) method and of k-folding. All of the cross-validation techniques are conducted within the training set (that is, they do not involve any observation in the test set). In leave-one-out, one of the cases is taken as verification set, the pandect is built on the remaining cases (the learning set), and its prognosis is checked on the unique case in the verification set, with this experiment being repeated for each case in the training set. In k-folding, the training set is partitioned randomly into k (for example, 2, 5, or 10) subsets; one of these subsets is then selected as the verification set, the pandect is constructed on the remainder of the training set (viewed as the learning set), and the prognosis of the pandect is checked on the verification set. This experiment is repeated k times, for each of the k possible selections of the verification set.

The entire calibration process is conducted only on the training set and it is intended to identify the best parameters to be used in the construction of the LAD models, and not to validate the LAD predictions (that process is described below).

#### Validation

Validation of the LAD results can be carried out in two ways. First, the predictions of the pandect built on the training set must be checked on the test set. This is the most frequently used validation method. In order to increase the reliability of the proposed pandect, an additional validation procedure can be applied. In this second validation procedure, a new dataset is created that consists of all of the observations in the original training and test sets. The second validation consists of the application of the usual cross-validation techniques (k-folding and/or leave-one-out) to this augmented dataset, using the parameters found at the calibration stage.

### Illustration with a demonstration model

The LAD method was trained and calibrated on the same training set of 78 samples used by van't Veer and coworkers [3]. The prognosis results for LAD were validated on the same test set of 19 samples used by van't Veer and coworkers. The samples in the test set were disregarded during the training procedure.

#### Support set selection

The LAD method starts with a pre-processing procedure for the selection of a significant support set of genes, on which the proposed prognostic system will be constructed. Because these systems are expected to have high accuracy, we restricted our study only to those 13,387 genes whose log-ratio measurements of fluorescence intensities are known for every single patient (that is, we eliminated those genes that include missing data). Part of our feature selection uses some statistical measures, and for this purpose we normalize the data by applying the following formula: x → (x - x_min)/(x_max - x_min).

After removing variables based on these measures, the original variables are reintroduced and a support set is determined. We recall that a support set consists of a subset of variables with the property that a model can build on them (not including any variable outside the support set), which can distinguish positive cases from negative ones.

In our dataset, from the set of 13,387 genes, using the method presented by Alexe and coworkers [46], we have extracted several support sets, including one consisting of six genes (Table 1), on which we shall build a 'demonstration model' (Table 2). Out of the six genes in the support set, one is involved in cell growth and three are enzymes [52].

Because of the simplicity of the demonstration model, we use it to illustrate the various concepts and procedures of LAD.

#### Binarization

We have used a simple binarization technique to replace the expression level of each gene by several binary (0–1) variables, simply indicating whether the expression level does or does not exceed certain thresholds. In order to achieve this, we introduced several cut points into the range of fluorescence intensities of each gene, dividing it into three zones (low, medium, and high). The cut points for each particular gene were defined in such a way that the number of cases in the training set having low, medium, or high expression levels for that gene should be approximately equal.

#### Pattern and model generation

In order to ensure high reliability of the patterns used in the demonstration model, we restricted our search to patterns of prevalence at least 15% (for the enhanced model we required the prevalences to be at least 20%). Furthermore, in order to maximize the explanatory power of the patterns detected, we restricted our search to patterns of degree 3 at most (that is, involving at most three genes). In this way, using the support set of six genes we have identified a pandect of 215 positive and 170 negative patterns and extracted from it the demonstration model consisting of only nine positive and seven negative patterns, as shown in Table 2.

Each row in Table 2 describes a pattern. The first entry in the row is the name of the pattern (for example, P1 in the first row describes the first positive pattern). The next six entries describe the defining conditions of that pattern (for example, P1 is described by the three conditions 'Gene NM_003239 ≤ -0.014', 'Gene NM_004035 > -0.106', and 'Contig15031_RC ≤ 0.055'). The last two entries indicate the positive and negative coverages (that is, the number of cases satisfying the defining conditions of the pattern) and prevalences (that is, proportion of positive, or negative, cases satisfying the defining conditions) of the pattern on the training set. For instance, P1 covers 22 of the 34 positive cases and none of the negative cases in the training set; therefore, its positive and negative prevalences on the training set are 64.7% and 0%, respectively.

#### Prognosis

The availability of the pandect makes it possible to classify new (that is, not yet seen) observations as being positive or negative. As a matter of fact, diagnosis and prognosis are perhaps the most important applications of LAD to biomedical problems. The most direct way to apply LAD to prognostic problems is to examine which patterns are displayed by a new case. If the case displays only positive patterns, then it is assigned a poor prognosis. Similarly, if it displays only negative patterns, then it is assigned a good prognosis. If the case does not display any pattern, then no prognosis can be assigned to it; it should be noted that this situation is extremely rare and did not occur at all in the present study. Finally, if a case displays both positive and negative patterns, then a simple weighting procedure is applied to determine whether the positive or the negative patterns are predominant. The weighting procedure consists simply of comparing the proportion of the displayed positive patterns in the set of all positive patterns contained in the model (pandect), with the analogous proportion of negative patterns.

To illustrate the way in which a model can be used to predict the positive (negative) nature of a 'new' patient, let us consider one having the following values of his six attributes appearing in the demonstration model: AF018081 = -0.029, NM_003239 = -0.013, NM_004035 = -0.17, Contig26768_RC = -0.033, Contig15031_RC = 0.132, and Contig27639_RC = -0.16. This patient will satisfy one (P5) of the nine positive patterns and five (N1, N2, N4, N5 and N6) of the seven negative patterns appearing in the demonstration model shown in Table 2. Therefore, the 'prognostic index' of this patient will be (1/9) - (5/7) = -38/63; because the prognostic index is negative, the model predicts this patient to be in the 'negative' class.

#### Validation of the demonstration model

The demonstration model has been validated in several ways. First, the direct application of the model to the training set of 78 observations resulted in 100% correct prediction of the positive or negative nature of each case. The application of the model to the test set of 19 cases resulted in a weighted accuracy of 81.6%, with 16 cases correctly predicted, one positive case predicted as negative, and two negative cases predicted as positive. Finally, 20 five-folding experiments on the training set resulted in a weighted average of 75.3% correct prediction, whereas 20 five-folding experiments on the combined training and test set (containing 97 cases) resulted in a weighted average of 76.2% accurate prediction.

## Results

### Prognostic system

We examined in the previous section a demonstration model, built on the support set consisting of the six genes shown in Table 1. Although the demonstration model provided high simplicity and accuracy, its small size (number of genes and patterns) makes it vulnerable to a lack of stability for small variations in the level of gene expression. It is therefore reasonable to build a more robust model using a larger support set (for example, the enhanced support set of 17 genes, shown in Table 3). It should be emphasized that this support set was obtained independently of the support set of the demonstration model, not by the addition of supplementary genes to the set, but by a bottom-up construction, which aimed at a solid separation of positive and negative cases; the two support sets are disjointed.

In this section we examine the enhanced model built on the 17 genes shown in Table 3. The functions of these genes, obtained from the DAVID database [52], are summarized in Table 3. Out of the 17 genes in the larger support set, one is involved in cell growth, nine are involved in cellular metabolism and one is involved in cellular adhesion [52].

Based on this 17-gene support set we constructed the "enhanced" model shown in Table 4, which consists of 20 positive and 20 negative patterns. It can be seen that the patterns are very robust, having prevalences of up to almost 56% in the positive case and above 34% in the negative case.

The classification provided by the enhanced model for the 34 patients with poor prognosis and the 44 patients with good prognosis makes no errors in the training set (weighted accuracy = 100%). More significantly, on the 19-case test set (which includes 12 positive and seven negative cases), the system makes only one error and classifies correctly all of the other cases; thus, the system's weighted accuracy is 92.9%. The only error is a type 2 one, and it is due to the incorrect classification of negative sample 119. The supplementary validation tests based on an additional series of 20 five-folding experiments on the combined dataset of 97 cases showed an average weighted accuracy of 81.7%.

### Significant biomarkers

Based on the frequency of inclusion of genes in the positive patterns, it can be seen that Contig65439 (chromosome 20 open reading frame 178) plays a significant role in determining a poor prognosis, because it appears in 10 of the 20 positive patterns of the model. Similarly, Contig 55574_RC (F-box protein 41) plays a significant role in determining good prognosis, because it appears in 11 of the 20 negative patterns of the model.

### Promoters and blockers

A gene with the property that an increase in the intensity level of its expression (while the expression levels of the all other genes remain unchanged) can sometimes worsen the prognosis, but can never improve it, will be called a 'promoter'. Similarly, a gene with the property that a decrease in the intensity level of its expression (while the expression levels of the all other genes remain unchanged) can sometimes improve the prognosis, but can never worsen it, will be called a 'blocker'. Clearly, not every gene is a promoter or a blocker.

The model can identify promoters and blockers in the following way. If every occurrence of a gene among the positive patterns imposes a lower bound on its expression level (that is, in all those patterns whose definition includes a condition concerning that gene, the condition is of the form 'the expression level of that gene is ≥ than a prescribed level'), while every occurrence of the same gene among the negative patterns imposes an upper bound of its expression level (that is, in all those patterns whose definition includes a condition concerning that gene, the condition is of the form 'the expression level of that gene is ≤ than a prescribed level'), then it can be concluded that an increase in the expression level of that gene (assuming that the expression levels of all the other genes remain unchanged) may have as a result the activation of more positive patterns and/or the deactivation of some negative ones. Therefore, an increase in the expression level of such a gene can only increase the chances of metastasis formation. Such a gene will be called a promoter.

Similarly, if every occurrence of a gene among the negative patterns imposes an upper bound on its expression level (namely, in all those patterns whose definition includes a condition concerning that gene, the condition is of the form 'the expression level of that gene is ≤ than a prescribed level'), while every occurrence of the same gene among the positive patterns imposes a lower bound of its expression level (namely, in all those patterns whose definition includes a condition concerning that gene, the condition is of the form 'the expression level of that gene is ≥ than a prescribed level'), then it can be concluded that an increase in the expression level of that gene (assuming that the expression levels of all the other genes remain unchanged) may have as a result the activation of more negative patterns and/or the deactivation of some positive ones. Therefore, an increase of the expression level of such a gene can only decrease the chances of metastasis formation. Such a gene will be called a blocker.

Using these definitions it is shown in Table 3 that genes NM_001756, AL080059, and Contig55574_RC are promoters, whereas genes NM_020974, Contig65439, Contig15031_RC, Contig41383_RC, and Contig63102_RC are blockers. The genes AF148505 and AL049689 also exhibit blocker characteristics although to a somewhat lesser extent; we view them as weak blockers.

### Special classes of positive cases

In order to discover special classes, we conducted a series of two-means clustering experiments of the positive observations, but they did not reveal the existence of any special subgroups of observations. However, using the pattern-based representation of the positive cases (as described in the Materials and method subsection Pattern space), two-means clustering revealed the existence of two very special classes of patients. Despite the random element present in the nature of the two-means clustering procedure, it transpired that in the 100 experiments we have carried out, the positive observations were repeatedly and consistently clustered into the same two subgroups, which are denoted below by P^+++ ^(consisting of patient numbers 48, 50, 51, 59, 66, 68, and 69) and P^+ ^(consisting of patient numbers 46, 52, 54, 55, 60, 62, 63, 73, and 78) respectively; these subgroups have the following distinctive properties

#### Cohesion

The seven patients belonging to P^+++ ^are assigned to a common cluster in 86% of the experiments, whereas the nine patients belonging to P^+ ^are assigned to another common cluster in 98% of the experiments.

#### Predictability

In each validation experiment of the prognostic system by the leave-one-out method, the prognosis of every single observation in P^+++ ^was correct; the 100% accuracy of the prognostic system on set P^+++ ^is much higher than its 82.3% accuracy on the set of positive cases not contained in P^+++^. On the other hand, the accuracy of predictions for the patients in class P^+ ^is only 55.6%.

#### Distinctive coverage by patterns

Each patient belonging to class P^+++ ^satisfies 50–90% of the positive patterns (68.5% on average), whereas each patient belonging to P^+ ^satisfies only 10–30% of the positive patterns (20% on average).

#### Distinctive gene expression ranges

The smallest interval of the 17-dimensional real space containing P^+++ ^does not contain any other positive or negative observation, whereas the one containing P^+ ^also contains seven negative observations (Table 5).

#### Statistical distinctions of clinical features

We shall say that feature 'f' is a 'contrastor' of subset S' of the positive cases from the complementary set S" (consisting of those positive cases that do not belong to S') if the following two conditions hold: the average value of f in S' does not belong to the 95% confidence interval of the values of f in S"; and the average value of f in S" does not belong to the 95% confidence interval of the values of f in S'. With this definition, it can be seen in Table 6 that the diameter and, to some extent, the grade are contrastors, which distinguish P^+++ ^from its complement in the positive class. It can be also observed (see Table 7) that class P^+ ^has some distinguishing characteristics (for example, the average PRp [progesterone receptor] of the patients in this class is 55.6, whereas the average PRp of the positive patients outside class P^+ ^is 27.6, with the 95% confidence interval ranging from 12.6 to 42.6).

#### Summary

It is clear that the classes P^+ ^and P^+++ ^are very special and that all of the characteristics listed above indicate that it is most likely that the patients belonging to class P^+++ ^have a very strong tendency toward developing metastases, whereas those in P^+ ^have a substantially reduced tendency.

### Special classes of negative cases

Using the pattern-based representation of cases described in the subsection Pattern space (above), we also carried out 100 two-means clustering experiments within the set of negative observations. Despite of the random element present in the nature of the two-means clustering procedure, it transpired that, similar to the positive class, the negative class also contains two disjointed (but not exhaustive) special subclasses. These are denoted below by N^--- ^(consisting of patient numbers 10, 18, 21, 23, 30, 32, 37, and 38) and N^- ^(consisting of patient numbers 2, 3, 4, 6, 8, 9, 11, 12, 13, 15, 16, 17, 19, 20, 22, 24, 26, 27, 28, 33, 34, 36, 39, 40, 41, and 44), respectively, and have the following distinctive properties.

#### Cohesion

The eight patients belonging to N^--- ^are assigned to a common cluster in 88% of the experiments, whereas the 26 patients belonging to N^- ^are assigned to a common cluster in 95% of experiments.

#### Predictability

In each validation experiment of the prognostic system by the leave-one-out method, the prognosis of every single observation in N^--- ^was correct; the 100% accuracy of the prognostic system on set N^--- ^is much higher than its 77.8% accuracy on the set of negative cases not contained in N^---^. On the other hand, the accuracy of predictions for the patients in class N^- ^is only 73.1%.

#### Distinctive coverage by patterns

Each patient belonging to the class N^--- ^satisfies 50–70% of the negative patterns (57.5% on average), whereas each patient belonging to N^- ^satisfies only 5–35% of the positive patterns (20% on average).

#### Distinctive gene expression ranges

The smallest interval of the 17-dimensional real space containing N^--- ^does not contain any other positive or negative observation, whereas the one containing N^- ^also contains eight positive observations (Table 8).

#### Statistical distinctions of clinical features

Similar to the positive case, we shall say that feature 'f' is a 'contrastor' of subset S' of the negative cases from the complementary set S" (consisting of those negative cases that do not belong to S') if the following two conditions hold: the average value of f in S' does not belong to the 95% confidence interval of the values of f in S"; and the average value of f in S" does not belong to the 95% confidence interval of the values of f in S'. With this definition, it can be seen in Table 9 that grade, estrogen receptor positive, and (to some extent) lymphocytic infiltrate are contrastors of N^---^. As far as class N^- ^goes, Table 10 shows the differences between the average values of some of the parameters in class N^- ^compared with average values of the same parameters in the set of negative cases outside N^-^.

#### Summary

It is clear that the classes N^- ^and N^--- ^are very special; all of the characteristics listed above indicate that it is most likely that patients belonging to class N^--- ^are very strongly resistant to development of metastases, whereas those in class N^- ^have a substantially milder resistance.

## Discussion

### Comparison of weighted accuracies

On the training set of 34 positive and 44 negative cases, the model reported by van 't Veer and coworkers [3] model misclassifies 12 positive and three negative cases. The proposed enhanced model classifies 100% of the cases in the training set correctly. On the 19-case test set, the van 't Veer model misclassifies two cases, whereas the enhanced model misclassifies one. We do not know whether the performance of the model presented by van 't Veer and coworkers [3] has been subjected to cross-validation (for example, by k-folding or leave-one-out experiments), and therefore we can not conduct a comparison with the cross-validation results of LAD, as shown in Table 11.

#### Comparison of support sets

The study by van 't Veer and coworkers [3] considered two support sets consisting of 70 and 231 selected genes, whereas the enhanced model in the present study used a support set of 17 genes. Accuracy in distinguishing cases of poor and good breast cancer prognosis provided by the subset of 70 genes selected by van 't Veer and coworkers was revalidated and confirmed by van de Vijver and colleagues [53] in a different cohort of patients.

In order to assess further the performance of the reported subsets of 231 and of 70 genes selected by van 't Veer and coworkers [3], and of the support set of 17 genes selected for the proposed enhanced LAD model, we applied LAD to each of these three subsets of genes. We then constructed separate predictive models on the training set and on the entire dataset (consisting of 78 and 97 samples, respectively), and tested their accuracy direct application both to the training set of 78 and to the entire dataset of 97 samples, and also by cross-validation, consisting of 20 five-folding experiments. The results are shown in Table 12.

Furthermore, we repeated the same type of experiments by comparing the weighted accuracies of applying five frequently used classification methods to the three support sets discussed above; these classification methods include artificial neural networks, support vector machines, logistic regression, nearest neighbours and decision trees, and are included in the publicly available software WEKA [54]. The results are given in Tables 13, 14, 15 and show that the average weighted accuracy of the five methods applied to the support set of 17 genes compares favourably with the results obtained using the two larger support sets of van 't Veer et al. and coworkers.

From these tables we can estimate the comparative average weighted accuracies of the different predictive models constructed on the 17 genes of the enhanced model, and on the 70 and 231 genes selected by van't Veer and coworkers [3]. It can be seen that the 95% confidence intervals of weighted accuracy estimated on the test set for the three predictive models that use 17, 70 and 231 genes were 59.20–77.65, 42.15–58.91 and 72.67–76.79, respectively. Clearly, we can conclude that the weighted accuracy in distinguishing patients with good and poor breast cancer prognosis is best for the model using 231 genes, is at a comparable (although slightly lower) level for the model using 17 genes, and is at a substantially lower level for the model using 70 genes.

#### Individual versus collective biomarkers

One of the important hypotheses raised by the LAD approach concerns the role played in an accurate prognostic system by those genes that have the greatest correlation with outcome. In contrast to the conventional approach, LAD aims to go beyond the straightforward goal of identifying genes with important individual contributions to distinguishing between breast cancer patients with good and poor prognosis, instead focusing on those genes that – taken as a group – have the greatest collective prognostic potential.

The breast cancer prognostic system developed in the present study confirms the hypothesis that the most accurate prognostic systems do not necessarily include only genes with strong correlations with outcome. Indeed, the 70 biomarkers used in the study by van 't Veer and coworkers [3] are extracted from the pool of 231 genes that (taken individually) are most highly correlated with the outcome. On the other hand, the 17-gene support set selected by LAD includes several genes whose correlation with the outcome in absolute value is very low. The average absolute value of Pearson correlation with the outcome of the 17 individual genes in the support set of the enhanced LAD model is only 0.33. However, the average absolute value correlation with the outcome of the 40 positive and negative patterns (which can be viewed as collective biomarkers) is higher, at 0.46.

It is interesting to note that the overlap between the set of 17 genes selected by LAD and the set of the 70 genes used in the study by van't Veer and coworkers [3] consists of only four genes (AL080059, NM_003748, NM_020974 and Contig63102_RC). Also, the overlap between the set of 17 genes and the pool of 231 genes, from which the 70 biomarkers were extracted by van't Veer and coworkers, consists of only eight genes (the four mentioned above and AB033007, AF148505, Contig42421_RC, and Contig37063_RC).

The high accuracy of the LAD model is not due to the role of the individual genes selected, but rather to the interactions among various genes in the 'collective biomarkers' represented by patterns. The concept of collective biomarkers is crucial to the LAD approach.

#### Contrast between training and test sets

One of the most frequently used validation techniques in a model learned on a training set is to apply it to a test set, and to compare the accuracies of the model's predictions on the two sets. It is usually assumed that characteristics of the training and test sets are very similar. The accuracy of predictions obtained by LAD and other machine learning methodologies on the test set is usually lower than that on the training set. This phenomenon can be easily explained by the fact that any such model learns the obvious and less obvious characteristics of the training set, not all of which may be represented in the test set. Surprisingly, in our analysis, the weighted accuracy on the test set (92.9%) turned out to be even higher than that estimated by cross-validation on the training set (82.5%). This suggests that a previously unrecognized, possibly substantial, difference existed between the training and the test sets. In fact, we determined that this is the case.

Indeed, it can be seen that for the set of all observations in the training set, with the exception of case number 70 (Sample 70), the intensity levels of gene NM_005839 (Ser/Arg-related nuclear matrix protein [plenty of prolines 101-like]) are consistently less than or equal to 0.19. On the other hand, on the test set the intensity levels of the same gene are consistently greater than 0.19. Therefore, it is clear that the intensity levels of gene NM_005839 distinguish completely the observations in the training set (with the exception of observation 70) from all the observations in the test set.

The above finding is made even clearer by considering patterns. It transpires that hundreds of patterns of degree 2 can be found that completely separate the training set and the test set, without any exceptions (not even for the observation 70 mentioned above).

The existence of pairs of genes that can distinguish between the training and test sets is an extremely rare situation. The existence of individual genes allowing such a distinction is clearly even more surprising. Even in datasets in which the training and test samples are collected in different laboratories, the existence of such genes or pairs of genes is highly unlikely. For instance, no such separation exists for the microarray dataset Leukemia AML-ALL studied by Golub and coworkers [55].

As an additional distinguishing characteristic of the training and test sets, let us consider the upper and the lower bounds of each variable for the 19 test cases, as shown in Table 16. It is clear that the measurements of none of the training set cases fit into the ranges of the 17 variables in the table. Technically, this means that if we define the interval closure of a set S of points as being the smallest interval [S] of the 17-dimensional Euclidean space R^17 ^spanned by the points in S, then the interval [test set] does not contain any of the observations included in the training set.

The observations presented above led us to the conclusion that the training and the test sets have different characteristics.

#### Individualized therapy

An important consequence of the identification of genes that are promoters or blockers is the possibility of targeting therapies in such a way that they should raise the expression of some blockers and/or lower those of some promoters. An even more attractive challenge is that of developing individualized therapies, which target the particular blockers and promoters present in the specific positive and negative patterns 'triggered' by the expression levels of an individual's genes.

#### Prognostic index

The results presented in the subsections Special classes of positive cases and Special classes of negative cases (above) indicate the existence of a possibly strong correlation between the weighted accuracy of prognosis and the proportion of patterns covering a case. Similarly to the index introduced by Alexe and coworkers [40] for risk stratification among cardiac patients, it is to be expected that a prognostic index for breast cancer patients could also be developed.

### Comparison with other studies

Various research groups have focused on finding molecular signatures of cancer prognosis. Ramaswamy and coworkers [56] analyzed the gene expression profiles of 12 metastatic adenocarcinoma nodules of diverse origins (lung, breast, prostate, colorectal, uterus, and ovary), and detected 17 gene signatures associated with metastases. Eight out of the 17 genes (*SNRPF *[small nuclear ribonucleoprotein F], EIF4EL3 [elongation initiation factor 4E-like 3], *HNRPAB *[heterogeneous nuclear ribonucleoprotein A/B], *DHPS *[deoxyhypusine synthase], *PTTG1 *[securin], *COL1A1 *[type 1 collagen α_1_], *COL1A2 *[type 1 collagen α_2_], and *LMNB1 *[lamin]) were found to be upregulated in metastases, whereas the remaining nine genes (*ACTG2 *[actin γ_2_], *MYLK *[myosin light chain kinase], *MYH11 *[myosin heavy chain 11], *CNN1 *[calponin 1], *HLA-DPB1 *[MHC class II DPβ1], *RUNX1 *[runt-related transcription factor 1], *MT3 *[metallothioein 3], *NR4A1 *[nuclear hormone receptor TR3], and *RBM5 *[RNA binding motif 5]) were found to be downregulated. None of these 17 genes was included among the poor prognosis signature in the study by van 't Veer and coworkers [3], whereas only one of them (*COL1A1*) was included in the support set of LAD.

Using cDNA gene expression profiling, van 't Veer and colleagues [57] showed that human primary breast cancer tumors are similar to distant metastases of the same patient. As concluded by Weigelt and coworkers [57], these findings support the finding of van 't Veer and coworkers [3] that the outcome of breast cancer can be predicted by the gene expression signature of primary tumors. We wished to determine whether the genes in the support set identified by LAD as being predictive of the breast cancer outcome would be able to distinguish between paired primary breast tumors and metastases. However, none of the genes in the LAD support set of 17 genes were used in the 18,336 cDNA microarrays of Weigelt and coworkers [57].

In a recent study, Dai and coworkers [58] showed that within a group of patients with high estrogen receptor there is a group of 50 mostly cell-cycle genes that are able to predict the occurrence of metastases. The LAD support set of 17 genes are disjointed with the set of 50 genes identified by Dai and coworkers [58].

The fact that gene expression signature is predictive of human breast cancer outcome was confirmed by various other independent groups. For example, in an earlier study, Bertucci and coworkers [59] confirmed that a 23-gene predictor set can distinguish between breast cancer tumors associated with different survival rates. In that study, the cDNA profiles of tumor samples from 55 women with poor prognosis breast cancer treated with adjuvant anthracycline-based chemotherapy were determined for 1000 candidate cancer genes. Those investigators were able to distinguish three classes with significantly different clinical outcome, as well as two estrogen receptor positive tumor subgroups with different survival rates. The 23 genes identified in that study that predicted survival of chemotherapy-treated patients were disjointed from the support set of 17 genes identified by LAD to predict metastases.

Sotiriou and coworkers [60] analyzed the cDNA expression profiles of 99 breast cancer tumors and identified 485 genes (out of the 7650 probe elements) associated with prognosis in breast cancer. Only 11 of the 485 genes were included in the subset of 231 genes identified by van 't Veer and coworkers [3] as being correlated with breast cancer outcome. Sotiriou and coworkers showed these 11 genes to be able to separate the breast cancer patients into two groups with different survival rates, thus confirming the findings of van 't Veer and colleagues. Of the 17 genes in the LAD enhanced support set, none were found in the set of 485 genes [60].

Sorlie and colleagues [61] re-evaluated the performance of the 231 genes of van 't Veer and coworkers in a comparative study involving data from two other previous independent groups (Norway/Stanford [62] and West and colleagues [63]). Out of the 231 genes identified by van 't Veer and colleagues, 77 were found in the Norway/Stanford dataset. Cross-validation experiments [61] showed that these 77 genes were able to discriminate between poor and good prognosis of breast cancer tumors presented by van 't Veer and colleagues [3] with 81% accuracy [3], thus confirming the findings of van 't Veer. In addition, Sorlie and coworkers [61] selected a subset of 534 intrinsic genes from the data in their previous report [62], refining the results of van 't Veer and coworkers [3] and West and colleagues [63], and showed the existence of several subtypes of tumors. Sorlie's list of 534 intrinsic genes and LAD's support set of 17 genes contain only one gene in common (ectonucleoside triphosphate diphosphohydrolase 2).

Paik and coworkers [64] identified a set of 21 genes (including 16 cancer-related and five reference genes) whose expression could be used to predict the distant recurrence of breast cancer in node-negative patients who were treated with tamoxifen. The genes were selected from among 250 candidate genes discussed in the literature and which had high correlation with the outcome of 447 patients in three independent clinical studies. The set of genes identified by Paik and coworkers and the genes identified by LAD were disjointed.

The fact that the vast majority of the 17 collective biomarkers identified by LAD do not appear in the support sets selected by univariate statistical methods clearly illustrates that combinatorial techniques can substantially supplement univariate statistical methods in discovering sets of significant genes; the set of clinically significant genes is far from being unique, because many – even disjointed – significant sets of genes have already been detected; and it is very likely that many as yet undiscovered sets of significant genes exist.

## Conclusion

Our LAD-based analysis of the data presented by van 't Veer and coworkers [4] identified a new support set of 17 genes that can fully distinguish between cases with poor prognosis and those with good prognosis. The selection of the set of 17 genes took into account their collective interactive role in distinguishing cancer cases from controls (namely, we did not simply select those genes that, taken individually, have particularly high expression levels or high correlations with the outcome). We also established an explicit and highly accurate classification model for breast cancer diagnosis, in which every decision is explicit and transparent (that is, fully described by the patterns of gene expression displayed by each individual patient). Furthermore, we identified the relative importance of each of the 17 genes, and identified those that have a blocking or contributing influence on breast cancer. A library of patterns (that is, logically synthesized combinations of gene expression patterns that act as biomarkers for prognosis of breast cancer in large proportions of the population) was established. Finally, we established a method for the automatic generation of patterns of gene expressions that have a determining effect on classification. The classification power of the patterns suggests the research hypothesis that they signify the existence of an underlying biologic mechanism, which requires elucidation.

This study suggests the applicability of the nonparametric combinatorial method of LAD to genomic analysis of other human cancers, as well as to the design of individualized therapies based on the specific patterns of gene expressions for each patient.

## Abbreviations

LAD = logical analysis of data.

## Competing interests

The authors declare that they have no competing interests.

## Authors' contributions

The authors made complementary contributions to this manuscript. All authors read and approved the final manuscript.

## Figures and Tables

**Table 1:** The six-gene support set of the demonstration model

Gene Index	Van't Veer id	GeneBank	DAVID gene name
1	AF 018081	AF018081	Collagen, type XVIII, alpha 1
2	NM 003239	NM_003239	Transforming growth factor, beta 3
3	NM 004035	NM_004035	Acyl-Coenzyme A oxidase 1, palmitoyl
4	Contig26768_RC	AI743607	Exostoses (multiple) 1
5	Contig15031_RC	AI347425	Oligodendrocyte myelin glycoprotein
6	Contig27639_RC	AW134837	Ectonucleoside triphosphate diphosphohydrolase 2

**Table 2:** Demonstration LAD model consisting of nine positive and seven negative patterns on the support set of six genes

Patterns	Definition of patterns						Patterns' coverages (prevalences) on training set	
			
	AF018081	NM_003239	NM_004035	Contig26768_RC	Contig15031_RC	Contig27639_RC		
	
	Attr. 1	Attr. 2	Attr. 3	Attr. 4	Attr. 5	Attr. 6	Positive prevalence	Negative prevalence
P1		≤-0.014	>-0.106		≤0.055		22 (64.71%)	0
P2		>-0.232, ≤0.0575	>-0.106			>-0.2305	17 (50%)	0
P3		≤-0.0945			≤0.0915	>-0.1555	13 (38.24%)	0
P4	>-0.12	>-0.1555, ≤-0.014			≤0.1145		12 (35.29%)	0
P5	>-0.12, ≤0.0055	≤0.0575			≤0.1485		11 (32.35%)	0
P6	>-0.2025		>-0.106, ≤0.0455		>-0.0065, ≤0.055		11 (32.35%)	0
P7	>-0.08			>-0.0345		>-0.1555, ≤0.1445	9 (26.47%)	0
P8	>0.071		>-0.106, ≤0.0775			>-0.1555	9 (26.47%)	0
P9		>-0.319		>0.035		>-0.1555, ≤0.1445	6 (17.65%)	0
N1	≤0.071			≤0.098	>0.0915		0	15 (34.09%)
N2					>0.1145	≤0.037	0	15 (34.09%)
N3	≤0.071	>0.0575	>-0.0635				0	14 (31.82%)
N4			≤-0.106				0	13 (29.55%)
N5		>-0.014				≤-0.1555	0	12 (27.27%)
N6				>-0.1335, ≤0.098	>0.055	≤0.037	0	11 (25%)
N7		>-0.319		>0.035	>0.055, ≤0.1485		0	7 (15.91%)

LAD, logical analysis of data.

**Table 3:** The 17-gene support set of the enhanced model.

Gene Index	Van't Veer id	GeneBank	DAVID_GENE_NAME
1	AB033007	AB033007	KIAA1181 protein
2	NM_001661	NM_001661	ADP-ribosylation factor 4-like
3	NM_001756	NM_001756	Serine (or cysteine) proteinase inhibitor, clade A (alpha-1 antiproteinase, antitrypsin), member 6
4	AF148505	AF148505	Aldehyde dehydrogenase 6 family, member A1
5	Contig42421_RC	AI912791	F-box protein 16
6	NM_003748	NM_003748	Aldehyde dehydrogenase 4 family, member A1
7	NM_020974	NM_020974	Signal peptide, CUB domain, EGF-like 2
8	AL080059	AL080059	TSPY-like 5
9	AL110129	AL110129	Mitochondrial ribosomal protein S22
10	Contig15031_RC	AI347425	Oligodendrocyte myelin glycoprotein
11	Contig65439	AI572600	Chromosome 20 open reading frame 178
12	Contig37063_RC	AA579843	Poly (ADP-ribose) glycohydrolase
13	Contig41383_RC	AA142876	Asparaginase like 1
14	AL049689	AL049689	Tenascin N
15	Contig63102_RC	AI583960	Hypothetical protein FLJ11354
16	Contig55574_RC	AA524093	F-box protein 41
17	Contig38451_RC	AA497035	Not available

**Table 4:** 'Enhanced LAD model' consisting of 20 positive and 20 negative patterns on support set of 17 genes

**Patterns**	**Definition of Patterns**																	**Patterns' coverages (prevalences) on training set**	
			
	AB033007	NM_001661	NM_001756	AF148505	Contig42421_RC	NM_003748	NM_020974	AL080059	AL110129	Contig15031_RC	Contig65439	Contig37063_RC	Contig41383_RC	AL049689	Contig63102_RC	Contig55574_RC	Contig38451_RC		
	
	Attr. 1	Attr. 2	Attr. 3	Attr. 4	Attr. 5	Attr. 6	Attr. 7	Attr. 8	Attr. 9	Attr. 10	Attr. 11	Attr. 12	Attr. 13	Attr. 14	Attr. 15	Attr. 16	Attr. 17	Pos Prev	Neg Prev
P1			>-0.42							≤0.09	≤0.06							19 (55.9%)	0
P2						≤0.07					≤-0.01		≤0.07					18 (52.9%)	0
P3			>-0.42						≤0.06		≤0.06							18 (52.9%)	0
P4											≤-0.01	≤0.38	≤0.07					18 (52.9%)	0
P5						≤0.07	≤-0.45				≤0.06							17 (50.9%)	0
P6	≤0.33										≤-0.01				≤-0.104			16 (47.1%)	0
P7						≤0.07					≤-0.01					>-0.02		16 (47.1%)	0
P8						≤0.07		>-0.295		≤0.033								16 (47.1%)	0
P9			>-0.42								≤0.06			≤-0.001				14 (41.2%)	0
P10		>-0.1			≤-0.11						≤-0.01							14 (41.2%)	0
P11		≤0.03					≤-0.45						≤0.19					13 (38.2%)	0
P12						≤0.07		>-0.295								>0.08		13 (38.2%)	0
P13		≤0.35						>-0.295								>0.08		13 (38.2%)	0
P14												≤0.3		≤-0.001		>0.08		13 (38.2%)	0
P15		≤-0.03											>-0.16, ≤0.07					12 (35.3%)	0
P16						≤0.35	>-0.96, ≤-0.7											10 (29.4%)	0
P17			>-0.22				≤0.055	>-0.295										10 (29.4%)	0
P18			>-0.22			>-0.48		>-0.1										10 (29.4%)	0
P19			>-0.22		≤0.32		≤0.055											10 (29.4%)	0
P20			>-0.22					>-0.1			>-0.27							10 (29.4%)	0
N1		>0.09													>-0.005			0	15 (34.1%)
N2								≤-0.295				>0.06				≤-0.02		0	15 (34.1%)
N3					>-0.11							≤0.14				≤-0.02		0	15 (34.1%)
N4							>0.055									≤-0.02	≤1.88	0	14 (31.8%)
N5													>0.07	>-0.001, ≤0.17				0	14 (31.8%)
N6							>0.055									≤-0.02		0	14 (31.8%)
N7			≤-0.22								>0.06				>-0.005			0	14 (31.8%)
N8							>0.055									≤-0.02		0	14 (31.8%)
N9		>0.09													>-0.005		>-0.083	0	14 (31.8%)
N10										>0.09						>-0.12, ≤0.08		0	13 (29.5%)
N11				>-0.03								>0.06, ≤0.14						0	13 (29.5%)
N12				>0.077		≤0.35	>0.055											0	13 (29.5%)
N13				>0.077					≤0.34							≤-0.0213		0	13 (29.5%)
N14			≤-0.22		≤0.18		>0.055											0	13 (29.5%)
N15	≤0.21			>0.077												≤-0.0213		0	12 (27.3%)
N16								≤-0.49								>-0.1207	≤1.877	0	12 (27.3%)
N17												>0.06, ≤0.14				≤-0.0213		0	12 (27.3%)
N18												≤0.22				>-0.12, ≤-0.02		0	12 (27.3%)
N19		≤0.16	≤-0.42				>-0.197											0	12 (27.3%)
N20						>-0.204	>-0.197				>0.13							0	11 (25.0%)

LAD, logical analysis of data

**Table 5:** Description of the cases in the special positive class P^+++^

	Gene Accession Number	AB033007	NM_001661	NM_001756	AF148505	Contig42421_RC	NM_003748	NM_020974	AL080059	AL110129	Contig15031_RC	Contig65439	Contig37063_RC	Contig41383_RC	AL049689	Contig63102_RC	Contig55574_RC	Contig38451_RC
P^+++^	Lower bound	-0.13	-0.123	-0.193	-0.362	-0.281	-0.372	-1.125	-0.066	-0.078	-0.077	-0.268	-0.193	-0.095	-0.242	-0.453	-0.369	-0.119
	Upper bound	0.108	0.044	0.381	0.116	-0.058	0.041	0.783	0.518	0.054	0.071	-0.009	0.116	0.07	0.115	0.048	0.525	0.268
Positive cases not in P^+++^	Lower bound	-0.174	-0.129	-0.708	-0.514	-0.601	-2	-1.337	-0.783	-2	-0.044	-0.263	-0.222	-0.567	-0.291	-0.345	-0.334	-0.147
	Upper bound	0.363	0.329	0.638	0.386	0.671	0.487	0.942	0.776	0.418	0.418	0.211	0.494	0.393	0.431	0.444	0.256	2

**Table 6:** Contrastors differentiating the positive cases in P^+++ ^from the positive cases outside P^+++^

		Diameter (mm)	Grade
P^+++^	Average	30.71	3.00
	CI (95%)	25.31	3.00
		36.12	3.00
Positive cases outside P^+++^	Average	22.67	2.81
	CI (95%)	20.11	2.67
		25.22	2.96

CI, confidence interval.

**Table 7:** Contrastors differentiating the positive cases in P^+ ^from the positive cases outside P^+^

		PRp
P^+^	Average	55.56
	CI (95%)	26.50
		84.61
Positive cases outside P^+^	Average	27.60
	CI (95%)	12.59
		42.61

CI, confidence interval; PRp, progesterone receptor.

**Table 8:** Description of the cases in the special negative class N^---^

	Gene Accession Number	AB033007	NM_001661	NM_001756	AF148505	Contig42421_RC	NM_003748	NM_020974	AL080059	AL110129	Contig15031_RC	Contig65439	Contig37063_RC	Contig41383_RC	AL049689	Contig63102_RC	Contig55574_RC	Contig38451_RC
N^---^	Lower bound	0.041	-0.112	-0.65	0.007	-0.126	0.059	-0.976	0.05	0.05	0.085	-0.266	0.062	-0.251	0.039	-0.022	-0.255	0.02
	Upper bound	0.21	0.228	0.166	0.453	0.386	0.675	-0.038	0.394	0.394	0.285	0.293	0.247	0.401	0.35	0.278	-0.024	0.303
Negative cases not in N^---^	Lower bound	-0.144	-0.106	-0.734	-0.294	-0.407	-1.253	-0.844	-0.214	-0.214	-0.115	-0.307	-0.179	-0.291	-0.21	-0.395	-0.343	-0.206
	Upper bound	0.345	0.443	1.135	0.363	0.521	0.881	0.311	0.477	0.477	0.273	0.293	0.433	0.482	0.455	0.335	0.22	0.323

**Table 9:** Contrastors differentiating the negative cases in N^--- ^from the negative cases outside N^---^

		Grade	ERp	Lymphocytic infiltrate
N^---^	Average	1.75	78.75	0.00
	CI (95%)	1.43	61.60	0.00
		2.07	95.90	0.00
Positive cases outside N^---^	Average	2.42	57.22	0.14
	CI (95%)	2.17	44.98	0.02
		2.67	69.46	0.25

CI, confidence interval; ERp, estrogen receptor.

**Table 10:** Contrastors differentiating the negative cases in N^- ^from the negative cases outside N^-^

		Follow-up time (years)	Grade	ERp	PRp	Lymphocytic infiltrate
N^-^	Average	8.16	2.58	47.31	36.92	0.19
	CI (95%)	7.28	2.31	32.62	23.19	0.04
		9.04	2.85	62.00	50.65	0.35
Negative cases outside N^-^	Average	9.48	1.89	81.11	56.94	0.00
	CI (95%)	8.20	1.58	71.23	40.61	0.00
		10.76	2.20	90.99	73.28	0.00

CI, confidence interval; ERp, estrogen receptor; PRp, progesterone receptor.

**Table 11:** Comparison of weighted accuracies of the van 't Veer Classifier and the enhanced LAD model

	Training set (78 cases)		Test set (19 cases)	Entire dataset (78 + 19 cases)
	
	Direct classification (%)	Cross-validation (%)	Direct classification (%)	Cross-validation (%)
Van 't Veer classifier [4]	83.6	Not reported	88.7	Not reported
Enhanced LAD model	100	82.52	92.86	81.74

LAD, logical analysis of data.

**Table 12:** Comparison of weighted accuracies of the LAD models constructed on three different support sets

Support set	Training set (78 cases)		Test set (19 cases)	Entire dataset (78 + 19 cases)
	
	Direct classification (%)	Cross-validation (%)	Direct classification (%)	Direct classification (%)
231 genes (van't Veer [4])	100.00	79.48	84.52	78.35
70 genes (van 't Veer [4])	99.26	75.43	84.52	74.06
Proposed support set of 17 genes	100.00	82.52	92.86	81.74

LAD, logical analysis of data.

**Table 13:** Weighted accuracies of various models constructed on the support set identified by LAD

Method	Support set of 17 genes (LAD)			
	
	Training set (78 cases)		Test set (19 cases)	Entire dataset (78 + 19 cases)
	
	Direct classification (%)	Cross-validation (%)	Direct classification (%)	Cross-validation (%)
Artificial neural networks (1 hidden layer)	100.00	76.55	84.21	78.65
Support vector machines (linear kernel)	87.18	76.43	63.16	77.27
Logistic regression	94.87	76.87	73.68	77.95
Nearest neighbors	100.00	80.55	63.16	76.34
Decision trees (C4.5)	96.15	67.48	57.90	67.01
95% CI	91.03–100	71.33–79.82	59.20–77.65	71.25–79.64

CI, confidence interval; LAD, logical analysis of data.

**Table 14:** Weighted accuracies of various models constructed on the support set of 70 genes identified by van 't Veer et al.

Method	Support set of 70 genes (van 't Veer [33])			
	
	Training set (78 cases)		Test set (19 cases)	Entire dataset (78+19 cases)
	
	Direct classification (%)	Cross-validation (%)	Direct classification (%)	Cross-validation (%)
Artificial neural networks (1 hidden layer)	100.00	80.16	42.11	71.65
Support vector machines (linear kernel)	96.15	82.01	57.90	77.03
Logistic regression	100.00	73.52	47.37	73.79
Nearest neighbors	100.00	71.58	63.16	71.77
Decision trees (C4.5)	96.15	60.49	42.11	61.89
95% CI	96.61–100	66.09–81.01	42.15–58.91	66.27–76.18

The 70-gene set was reported by van 't Veer and coworkers elsewhere [4]. CI, confidence interval; LAD, logical analysis of data.

**Table 15:** Weighted accuracies of various models constructed on the support set of 231 genes identified by van 't Veer and coworkers

Method	Support set of 231 genes (van 't Veer [33])			
	
	Training set (78 cases)		Test set (19 cases)	Entire dataset (78 + 19 cases)
	
	Direct classification (%)	Cross-validation (%)	Direct classification (%)	Cross-validation (%)
Artificial neural networks (1 hidden layer)	100.00	72.24	73.68	73.96
Support vector machines (linear kernel)	100.00	72.79	73.68	74.88
Logistic regression	100.00	71.21	73.68	75.63
Nearest neighbors	100.00	72.94	78.94	77.15
Decision trees (C4.5)	97.44	60.70	73.68	66.64
95% CI	98.48–100.00	65.39–74.56	72.67–76.79	70.07–77.24

The 70-gene set was reported by van 't Veer and coworkers elsewhere [4]. CI, confidence interval; LAD, logical analysis of data.

**Table 16:** Interval containing all the 19 cases in the test set and none of the 78 cases in the training set

	Gene Accession Number	AB033007	NM_001661	NM_001756	AF148505	Contig42421_RC	NM_003748	NM_020974	AL080059	AL110129	Contig15031_RC	Contig65439	Contig37063_RC	Contig41383_RC	AL049689	Contig63102_RC	Contig55574_RC	Contig38451_RC
Interval	Lower bound	-0.212	-0.227	-0.541	-0.268	-0.301	-0.295	-1.085	-0.606	-0.144	-0.282	-0.325	-0.307	-0.106	-0.219	-0.347	-0.325	-0.245
	Upper bound	0.187	0.017	0.29	0.22	0.394	0.309	0.557	0.401	0.241	0.062	0.303	0.272	0.117	0.304	0.331	0.576	0.183
